# A Review of the Design and Performance of Catalysts for Hydrothermal Gasification of Biomass to Produce Hydrogen-Rich Gas Fuel

**DOI:** 10.3390/molecules28135137

**Published:** 2023-06-30

**Authors:** Kapil Khandelwal, Philip Boahene, Sonil Nanda, Ajay K. Dalai

**Affiliations:** 1 Department of Chemical and Biological Engineering, University of Saskatchewan, Saskatoon, SK S7N 5A9, Canada; kak368@mail.usask.ca (K.K.); peb225@mail.usask.ca (P.B.); 2 Department of Engineering, Faculty of Agriculture, Dalhousie University, Truro, NS B2N 5E3, Canada; sonil.nanda@dal.ca

**Keywords:** biofuels, biomass, catalysts, cellulose, gasification, hemicellulose, hydrogen, lignin, methane, supercritical water

## Abstract

Supercritical water gasification has emerged as a promising technology to sustainably convert waste residues into clean gaseous fuels rich in combustible gases such as hydrogen and methane. The composition and yield of gases from hydrothermal gasification depend on process conditions such as temperature, pressure, reaction time, feedstock concentration, and reactor geometry. However, catalysts also play a vital role in enhancing the gasification reactions and selectively altering the composition of gas products. Catalysts can also enhance hydrothermal reforming and cracking of biomass to achieve desired gas yields at moderate temperatures, thereby reducing the energy input of the hydrothermal gasification process. However, due to the complex hydrodynamics of supercritical water, the literature is limited regarding the synthesis, application, and performance of catalysts used in hydrothermal gasification. Hence, this review provides a detailed discussion of different heterogeneous catalysts (e.g., metal oxides and transition metals), homogeneous catalysts (e.g., hydroxides and carbonates), and novel carbonaceous catalysts deployed in hydrothermal gasification. The article also summarizes the advantages, disadvantages, and performance of these catalysts in accelerating specific reactions during hydrothermal gasification of biomass, such as water–gas shift, methanation, hydrogenation, reforming, hydrolysis, cracking, bond cleavage, and depolymerization. Different reaction mechanisms involving a variety of catalysts during the hydrothermal gasification of biomass are outlined. The article also highlights recent advancements with recommendations for catalytic supercritical water gasification of biomass and its model compounds, and it evaluates process viability and feasibility for commercialization.

## 1. Introduction

Owing to increased growth in the population as well as urban and industrial development, global energy consumption has witnessed a dramatic rise over the years. Currently, 80% of the global energy demand is met by fossil fuels such as coal, natural gas, gasoline, and diesel. It cannot be denied that fossil fuels have long-term adverse effects on the environment and ecosystems, including global warming, an increase in greenhouse gas emissions, acid rain, and changes in weather patterns, to name a few [1]. On a global scale, CO_2_ emissions from the usage of fossil fuels such as coal, crude oil, and natural gas amount to 15, 12, and 8 billion tons, respectively [2]. Gradually phasing away from fossil fuels and seeking alternative and renewable sources of energy are urgently required.

Biofuels produced from renewable sources such as lignocellulosic biomass, livestock manure, microalgae, municipal solid waste, and sewage sludge are desirable alternatives to fossil fuels for meeting future energy demands and reducing carbon emissions [3,4]. Hydrogen (H_2_) has proven to be a clean alternative source to fossil fuels for meeting energy demands because of its zero carbon emissions, higher heating value of 140 MJ/kg, and adiabatic flame temperature of approximately 2100 °C. The combustion products of H_2_ are water and heat energy, compared to the combustion of fossil fuels which emits greenhouse gases such as CO_2_, CO, CH_4_, SO_x_, and NO_x_. In addition to being considered the fuel of the future, hydrogen is also utilized in a wide variety of commercial applications such as fuel cells, upgrading crude oil, synthesis of fine chemicals, metallurgy, pharmaceuticals, and the aerospace industry [5]. A main advantage of H_2_ is its ability to produce clean electricity through fuel cells [6]. H_2_ is also a valuable precursor in the production of various commodity and specialty chemicals, such as methanol, ammonia, alcohol, and aldehydes, through various catalytic and non-catalytic thermochemical conversion processes [7,8]. H_2_ is also extensively used by refineries in hydrotreating processes such as hydrodeoxygenation [9], hydrodenitrogenation [10], hydrodesulfurization [10], and hydrodemetallization [11] to upgrade crude oil and bio-oil to transportation-grade fuels. The sustainable nature of H_2_ and its increasing demand in many industrial and commercial sectors has entrenched it as an integral component of the circular economy.

**Figure 1:**
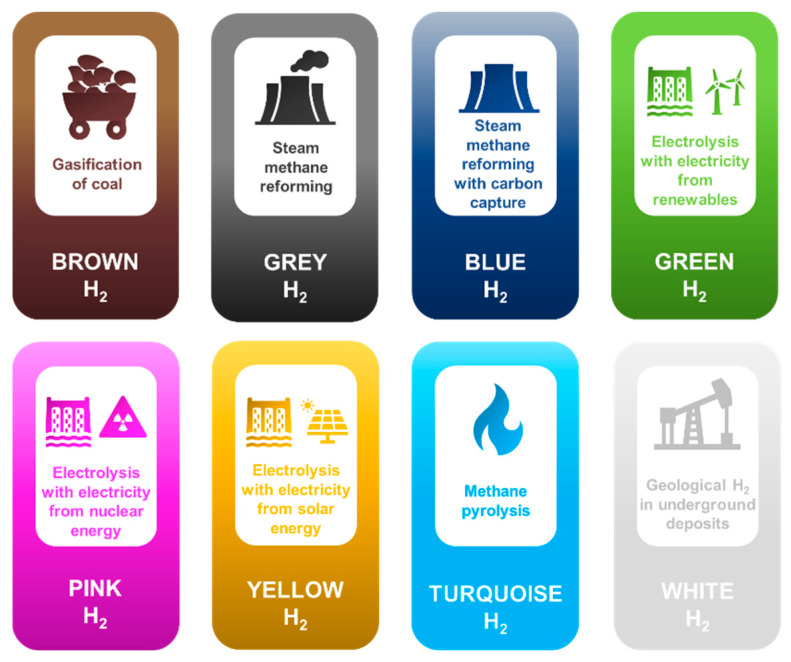
Different color shades of the hydrogen spectrum.

Although hydrogen gas has no color, its production routes have designated it different colors categorization. Hydrogen can be categorized as brown, grey, blue, green, pink, yellow, turquoise, and white based on its production from a wide variety of sources and technologies (Figure 1). Based on the source and production technology employed, hydrogen can be classified into different colors such as brown H_2_ (gasification of coal), grey H_2_ (steam reforming of methane), blue H_2_ (steam reforming of methane with carbon capture), green H_2_ (electrolysis using electricity from renewables), pink H_2_ (electrolysis using electricity from nuclear energy), turquoise H_2_ (methane pyrolysis), yellow H_2_ (electrolysis using electricity from solar power), and white H_2_ (geological H_2_ in underground deposits) [12].

Currently, the major route for the synthesis of H_2_ is the steam reforming of methane, which contributes to approximately 95% of global H_2_ production [13]. Nearly 250,000 standard cubic feet of CO_2_ is emitted per 1 million standard cubic feet of H_2_ produced from the steam reforming of CH_4_ [14]. Despite the significantly larger carbon footprint of the steam reforming of methane process, it is still commercially applied today. Although not widely commercialized, several sustainable pathways for hydrogen production from alternative sources are also available, such as electrolysis, photocatalysis, hydrothermal gasification, dark fermentation, and photo-fermentation [15].

The hydrothermal gasification conversion route is capable of sustainably producing H_2_ via renewable lignocellulosic biomass sources. This process utilizes water at either subcritical or supercritical conditions as a green solvent and reaction medium to disintegrate complex organic substrates to gases such as H_2_, CH_4_, CO, and CO_2_ [16]. When the temperature and pressure of water exceed its critical points of 374 °C and 22.1 MPa, respectively, supercritical water (SCW) is generated [17]. On the other hand, water is transformed into subcritical water when the temperature and pressure of water are slightly below or near its critical points. 

SCW experiences a significant change in its properties compared to liquid water at room conditions, imparting unique properties such as faster kinetics, a non-polar nature, and excellent solubility of gaseous molecules with the absence of interphase transfer boundaries [18,19]. Due to these versatile properties, supercritical water gasification (SCWG) can convert recalcitrant feedstocks with high moisture content into gaseous fuels enriched with combustible gases such as H_2_ and CH_4_. SCWG also does not require biomass drying because of its aqueous reaction medium, making the process energy efficient [20]. Due to these advantages, SCWG has recently gained popularity as an environmentally friendly process to produce H_2_ from waste feedstocks. 

The main products of SCWG are gases (e.g., H_2_, CO, CO_2_, CH_4_, and C_2+_), hydrochar, and liquid effluents. Hydrochar is a carbon-rich solid product resulting from depolymerization, dehydrogenation, decarboxylation, deamination, and aromatization of the organic feedstock used in SCWG [21]. Further activation and functionalization of hydrochar can enhance its surface area and properties for a wide variety of applications, such as solid fuel, adsorbent, catalyst support, activated carbon, carbon sequestration product, reinforcing material for composites, and soil amendment agent [22,23,24]. The liquid effluents resulting from the hydrothermal decomposition of biomass contain alcohols, furfurals, carboxylic acids, esters, ethers, aliphatics, aldehydes, ketones, and phenolics [25]. Some of these degradation compounds may further polymerize to form tar, which is a challenging component that causes plugging as well as heat and mass transfer limitations in the processors [26]. It should be noted that process conditions such as temperature, reaction time, pressure, and feedstock concentration largely impact the yields and composition of gases, liquids, and hydrochar from the SCWG of biomass [20].

Catalysts also play an important role in improving the process efficiencies of SCWG, especially carbon gasification efficiency and selectivities of gases, by regulating specific reaction mechanisms [27,28]. Several homogeneous and heterogeneous catalysts have been designed and investigated for the SCWG of different biomasses. However, the literature on the application of different catalysts in SCWG appears to be scattered. In addition, in-depth knowledge is scarce on understanding the different reaction pathways, mechanisms, and product properties impacted by homogeneous and heterogeneous catalysts in SCWG. Hence, this review paper attempts to categorically summarize the recent advancements in different homogeneous and heterogeneous catalysts used in SCWG. Furthermore, the challenges and shortcomings of different catalysts are also identified, followed by a discussion and recommendations for the effective design of catalysts, catalytic supports, and promoters used in the SCWG of biomass to produce high-value gaseous fuels.

## 2. Homogeneous Catalysts Used in Hydrothermal Gasification

Homogeneous catalysts used in SCWG generally consist of alkali metal and hydroxide catalysts. Table 1 summarizes some notable studies on the application of homogeneous catalysts in the SCWG process [29,30,31,32,33,34,35]. Homogeneous catalysts promote water–gas shift reactions by favoring C–C bond breakup, thus improving H_2_ yields [36]. The water–gas shift reaction results in the formation of H_2_ and CO_2_ because of the reaction of CO and H_2_O. The produced H_2_ can further react with the reactive intermediates generated by the catalytic action of homogeneous catalysts to increase overall gas yields [31]. Homogeneous catalysts usually have rapid conversion rates and can be used in both batch and continuous reactors. Homogeneous catalysts are also cost-effective with negligible sintering [26]. 

Su et al. [37] reported a base-catalyzed mechanism of alkali metals that enabled the water–gas shift reaction. The degradation intermediates were anions comprising hydroxides, carbonates and formates. Mixing the carbonates in water produced CO_2_ and hydroxides. Hydroxides can further combine with CO to produce formate. Further decomposition of formaldehyde can generate H_2_. Watanabe et al. [38] reported an ionic-catalyzed mechanism of alkali metals in the SCWG of methanol. They proposed that the ionic species stabilized the methanol by protonation or disassociation. Methanol then oxidized into CO, and protons stabilized the produced CO. CO_2_ was formed via the oxidation of CO, and hydroxide ions favored the water–gas shift reaction to convert CO into CO_2_. Thus, the oxidization of CO to CO_2_ was enhanced by the hydroxyl ions. 

**Figure 2:**
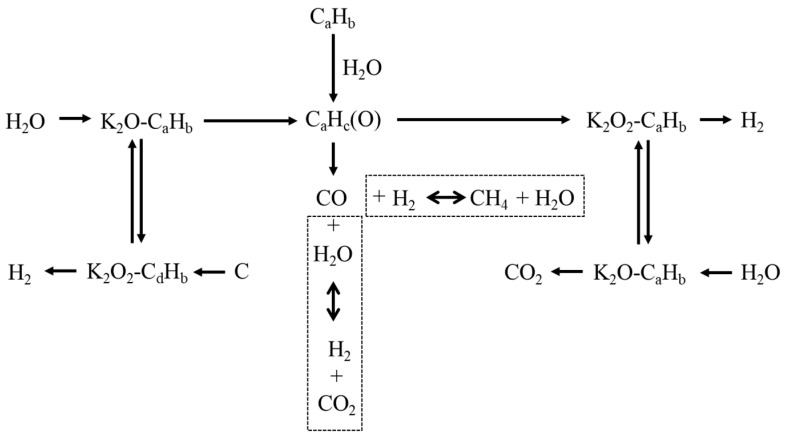
Catalytic mechanism of potassium in SCWG of biomass (adapted with permission from Ge et al. [39]).

Figure 2 represents a simplified catalytic mechanism of potassium metal in the SCWG of biomass [39]. Sınaǧ et al. [32] compared K_2_CO_3_ (a homogeneous catalyst) with Raney nickel (a heterogeneous catalyst) in the SCWG of glucose. Their results showed that the catalytic action of K_2_CO_3_ enhanced H_2_ production while suppressing the formation of phenols for improved gasification efficiency. K_2_CO_3_ demonstrated superior catalytic activity as compared to Raney nickel. K_2_CO_3_ showed higher yields of H_2_ and CO_2_ than Raney nickel, which confirmed its catalytic action to promote the water–gas shift reaction. The catalytic mechanism of K_2_CO_3_ in enhancing water–gas shift via formate (HCOO^−^K^+^) formation is presented in the following equations. The produced potassium formate further reacts with excess water to generate H_2_ with KHCO_3_, which decomposes into CO_2_ and K_2_CO_3_.

K_2_CO_3_ + H_2_O → KHCO_3_ + KOH (1)

HCOOK + H_2_O → KHCO_3_ + H_2_ (2)

2KHCO_3_ → CO_2_ + K_2_CO_3_ + H_2_O (3)

Madenoğlu et al. [30] studied the kinetics effects of K_2_CO_3_ in the SCWG of cellulose, lignin, and their mixtures. Their results showed that K_2_CO_3_ promoted the rates of gasification reactions and prevented the formation of char. Both gas and aqueous phase yields increased at the expense of char yield due to the catalytic effects of K_2_CO_3_. K_2_CO_3_ also favored the water–gas shift reaction, thus increasing the H_2_ yield.

Sınaǧ et al. [40] studied the catalytic effect of K_2_CO_3_ on glucose, phyto-mass (plant residues without proteins), and zoo-mass (meat residues containing proteins). The addition of K_2_CO_3_ had a significant influence in promoting the water–gas shift reaction during the SCWG of glucose and enhanced H_2_ production. However, its catalytic effects in promoting water–gas shift during the SCWG of phyto-mass and zoo-mass were minimal. 

Nanda et al. [35] compared four different homogeneous catalysts, Na_2_CO_3_, K_2_CO_3_, NaOH, and KOH, in the SCWG of Timothy grass. An increase in catalyst loading from 1% to 3% increased the total gas yield, as well as H_2_, CH_4,_ and CO_2_ yields, but decreased the CO yield for all catalysts. This indicated the catalytic action of alkali catalysts promoted gasification efficiency and the water–gas shift reaction. KOH showed the highest H_2_ yield of 9 mol/kg, followed by K_2_CO_3_, NaOH, and Na_2_CO_3_. A similar trend was observed for total gas yields. The highest total gas and H_2_ yields with KOH were explained by its catalytic action to promote the water–gas shift reaction. On the other hand, NaOH enhanced the methanation reaction with the consumption of H_2,_ increasing CH_4_ yields. Nanda et al. [41] also confirmed the superior catalytic effects of KOH in the SCWG of fructose where KOH showed a higher H_2_ yield than NaOH with nearly three times more H_2_ yield than non-catalytic reactions. 

Yanik et al. [42] compared the activities and selectivities of K_2_CO_3_, Trona, red mud, and Raney nickel catalysts in the SCWG of cotton stalk, corncob, and tannery wastes. Their results showed that all four catalysts significantly enhanced H_2_ yields by favoring water–gas shift and reforming reactions. K_2_CO_3_ demonstrated the highest H_2_ yield with no CO detected in the gas products. However, the catalytic activity of Trona was analogous to that of K_2_CO_3_. Ferreira-Pinto et al. [43] investigated the effects of NaOH, KOH, and Na_2_CO_3_ catalysts in the SCWG of lactose. The increase in H_2_ yield was highest with NaOH, followed by Na_2_CO_3_ and KOH. All catalysts inhibited char formation and significantly reduced the total organic carbon content in the reactants, indicating high gasification efficiencies.

Alkali catalysts can also significantly reduce the sulfur content in gas products. High sulfur content in gas products is a serious issue as its combustion can release SO_x_. Sulfur can also deactivate and poison the catalysts as well as corrode pipelines. Feng et al. [34] used different homogeneous catalysts (e.g., KOH, K_2_CO_3_, NaOH, Na_2_CO_3_, and activated carbon or AC) in the SCWG of sewage sludge. K_2_CO_3_ showed the best desulfurization effect and limited the H_2_S and SO_2_ contents to around 140 ppm and 200 ppm, respectively. The order of desulfurization effects of catalysts was found to be: K_2_CO_3_ > Na_2_CO_3_ > NaOH > KOH > AC. KOH demonstrated the highest H_2_ yield and selectivity. Alkali catalysts converted the unstable sulfur compounds into stable sulfur compounds by promoting cyclization and oxidation reactions, thus preventing the migration of sulfur into gas and liquid products. 

Zhong et al. [44] investigated the catalytic performance of KOH, K_2_CO_3_, KMnO_4_, and H_2_O_2_ on polycyclic aromatic hydrocarbons (PAHs) and gas formation during the SCWG of coking sludge. Their results showed that the PAH content decreased in the catalytic SCWG experiments. The catalytic action of KOH was attributed to its ability to promote free radical reactions during SCWG. These free radicals promote ring-opening reactions of PAHs, leading to their decomposition. KOH led to a higher H_2_ yield than K_2_CO_3_ because of an improved water–gas shift reaction through the formation of a formate intermediate and hydroxyl ions. These hydroxyl ions efficiently capture CO_2_ produced from the water–gas shift reaction. This shifted the equilibrium of the water–gas shift reaction towards the products side, thus producing more H_2_. Despite the numerous advantages of homogeneous catalysts, they can easily cause reactor plugging and corrosion in the reactor [45]. The recovery of homogeneous catalysts is also difficult compared to that of heterogeneous catalysts, which adds to overall process expenditures [46].

## 3. Heterogeneous Catalysts Used in Hydrothermal Gasification

Heterogeneous catalysts applied in the SCWG process can be broadly divided into two categories, namely metal oxides and transition metals. The recovery and recycling of heterogeneous catalysts are relatively easier compared to those of homogeneous catalysts [47]. Heterogeneous catalysts are more active, resulting in efficient and improved gasification efficiency [48]. They are also more selective for specific products by promoting desired reactions. A summary of promising studies on the use of heterogeneous catalysts in SCWG is presented in Table 2 [27,49,50,51,52,53,54,55]. 

### 3.1. Transition Metals

#### 3.1.1. Nickel-Based Catalysts

Nickel-based catalysts are the most widely used heterogeneous catalysts in SCWG because of their high activity compared to other expensive transition metal catalysts. Ni-based catalysts require comparatively lower temperatures and promote biomass gasification with higher efficiency. However, Ni-based catalysts can also consume the produced H_2_, CO, and CO_2_ due to their high methanation activity, producing CH_4_ [56]. Furusawa et al. [57] used the Ni/MgO catalyst in the SCWG of lignin. They studied its regenerative capabilities by recovering and reusing the catalyst thrice. The catalyst showed satisfactory regenerative capability before suffering from deactivation due to the formation of carbon and Mg(OH)_2_.

Zhang et al. [58] studied the SCWG of glucose and compared the activities and H_2_ selectivities of Ni, Co, Ru, and Cu transition metals on γ-Al_2_O_3_, AC, and ZrO_2_ supports. Both 10%Ni/γ-Al_2_O_3_ and 10%Ru/Al_2_O_3_ demonstrated the highest catalytic activities and H_2_ selectivities. The order of activity of the supports for the Ni catalyst was: γ-Al_2_O_3_ > ZrO_2_ > AC. Due to satisfactory results with 10%Ni/γ-Al_2_O_3_, further enhancement with Na, K, Mg, and Ru promotors was also studied. The addition of the 0.5%K promoter on 10%Ni/γ-Al_2_O_3_ significantly increased the H_2_ yield by favoring the water–gas shift reaction. 

Azadi et al. [28] studied the SCWG of various lignocellulosic feedstocks (e.g., glucose, fructose, cellulose, pulp, xylan, bark, and lignin) using five transition metals catalysts (e.g., Ni/Al_2_O_3_, Ru/C, Raney nickel, Ni/hydrotalcite, and Ru/Al_2_O_3_). The activities of Ni/Al_2_O_3_ and Ni/hydrotalcite catalysts for SCWG demonstrated the highest H_2_ selectivities. In contrast, Raney nickel showed the lowest H_2_ selectivity. Ni/α-Al_2_O_3_ and Ni/hydrotalcite also demonstrated low CH_4_ yields at high temperatures and longer reaction times. The high H_2_ selectivities of Ni/α-Al_2_O_3_ and Ni/hydrotalcite were attributed to the lower nickel dispersion and large crystallite sizes of Ni/α-Al_2_O_3_ and Ni/hydrotalcite catalysts compared to Raney nickel. The high nickel dispersion of Raney nickel strongly favored C–O bond cleavage compared to Ni/Al_2_O_3_ and Ni/hydrotalcite catalysts, thus explaining the low H_2_ selectivity of Raney nickel. The authors also reported that among all feedstocks, lignin was the most resistant to SCWG because of its branched polymeric structure. The lowest gas yield obtained from lignin was attributed to potential deactivation of the catalysts due to its sulfur content. 

Azadi et al. [27] compared Ni catalysts on different support materials, including γ-Al_2_O_3_, α-Al_2_O_3_, activated carbon, carbon nanotubes (CNT), hydrotalcite, MgO, SiO_2_, silica gel, TiO_2_, ZrO_2_, and various zeolites in the SCWG of glucose. The 20%Ni/α-Al_2_O_3_ catalyst showed the highest H_2_ selectivity, and Ni/CNT demonstrated high H_2_ yields (17–24 mmol/g) and high stability with maximum carbon gasification efficiency. On the other hand, Ni/MgO demonstrated a better H_2_ yield (26 mmol/g) and satisfactory carbon gasification efficiency. Due to its low cost and high stability, the authors further investigated the Ni/α-Al_2_O_3_ catalyst by varying Ni loading and using promoters. Tin increased the H_2_ selectivity but decreased the catalytic activity, whereas alkali promoters increased the carbon gasification efficiency but decreased the H_2_ selectivity. Lu et al. [50] also studied Ni-based catalysts with various promoted Al_2_O_3_ supports (e.g., CeO_2_/Al_2_O_3_, MgO/Al_2_O_3_, La_2_O_3_/Al_2_O_3_, and ZrO_2_/Al_2_O_3_) in the SCWG of glucose. CeO_2_/Al_2_O_3_ showed the highest H_2_ yield, followed by La_2_O_3_/Al_2_O_3_, ZrO_2_/Al_2_O_3_, Al_2_O_3_, and MgO/Al_2_O_3_. 

Onwudili and Williams [53] investigated the catalytic SCWG of various plastic wastes with Ru and Ni catalysts. By increasing RuO_2_ loading up to 5 wt% in the SCWG of low-density polyethylene, the H_2_ yield rose from 1 to 9.9 mol/kg at 450 °C in 1 h. However, the subsequent increase in RuO_2_ loading from 5 wt% to 20 wt% decreased the H_2_ yield to 4.9 mol/kg while increasing the hydrogen gasification and carbon gasification efficiency. By using a 20 wt% RuO_2_-γ-Al_2_O_3_ catalyst, polypropylene produced a high H_2_ yield and the highest carbon gasification efficiency of 99%. High- and low-density polyethylenes also showed similar gas yields, whereas polystyrene produced the lowest yields of C_2_-C_4_ gases. Low-density polyethylene demonstrated the highest H_2_ yield, followed by polystyrene, polypropylene, and high-density polyethylene. 

**Figure 3:**
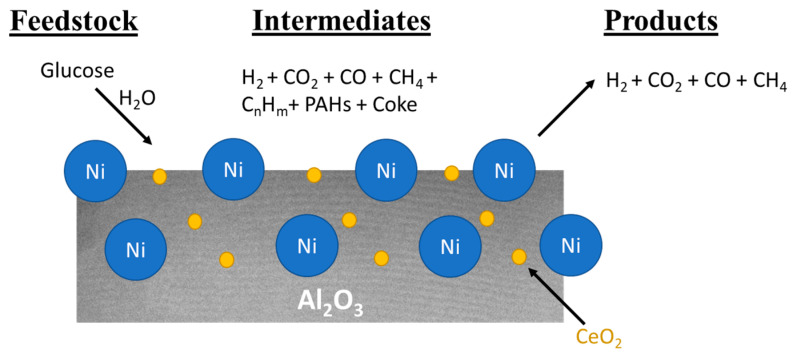
Catalytic mechanism of Ni/Ce-Al_2_O_3_ in SCWG of glucose (adapted with permission from Adamu et al. [59]).

Adamu et al. [59] studied Ce-mesoAl_2_O_3_ support impregnated with Ni in the SCWG of glucose (Figure 3). Ce-mesoAl_2_O_3_ had superior support properties compared to γ-Al_2_O_3_, such as moderate acidity, which helped to reduce coke formation and enabled high metal loading with low agglomeration. The Ni(20)/Ce-Al_2_O_3_ catalyst exhibited a very high H_2_ yield of 10.2 mol/mol of glucose. The meso-form led to the cracking of large intermediates such as tar compounds. Furthermore, Ce helped to improve the thermal stability of the alumina support.

Lu et al. [51] compared Ni, Cu, and Fe transition metals supported on MgO in the SCWG of wheat straw. The H_2_ yields varied with the application of different catalysts in the following order: Ni/MgO > Fe/MgO > Cu/MgO. Due to excellent H_2_ selectivity with Ni, the authors explored various supports, such as basic oxides (MgO and ZnO), acidic oxide (Al_2_O_3_), and amphoteric oxide (ZrO_2_). The H_2_ selectivities of Ni-supported catalysts varied in the order of Ni/MgO > Ni/ZnO > Ni/ Al_2_O_3_ > Ni/ZrO. Although the type of support had a minimal effect on H_2_ yield, a significant effect was observed on the decrease in CO yield. Basic oxide supports such as MgO and ZnO favored water–gas shift reactions, thus increasing H_2_ yields. The acidic support such as Al_2_O_3_ did not enhance the water–gas shift reaction. Hence, Ni/Al_2_O_3_ showed nearly double the CO yield as compared to the Ni/ZnO and Ni/MgO catalysts.

Okolie et al. [54] performed the SCWG of soybean straw using different Ni-based catalysts, catalyst supports, and promoters. ZrO_2_ and Al_2_O_3_ proved to be the most effective supports for Ni-based catalysts. Both 10%Ni-ZrO_2_ and 10%Ni-Al_2_O_3_ demonstrated higher H_2_ yields than other catalyst supports (e.g., CNT, SiO_2_/Al_2_O_3_, SiO_2_, and AC). Therefore, the authors further studied the effects of K, Na, and Ce promotors on Ni-based catalysts supported by ZrO_2_ and Al_2_O_3_. The 10%Ni-1%Ce/ZrO_2_ catalyst demonstrated the highest H_2_ yield of 10.9 mmol/g, followed by 10%Ni-1%K/ZrO_2_ and 10%Ni-1%Na/ZrO_2_. The relative increment in H_2_ yield and total gas yield without using any promoters was more substantial with the Ce and K promotors than with the Na promotor. However, the Na promotor showed the highest H_2_ yield with the Al_2_O_3_ support compared to the K and Ce promotors. The 10%Ni-1%Na/Al_2_O_3_ catalyst demonstrated the highest H_2_ yield (10.8 mmol/g) compared to 10%Ni-Ce/Al_2_O_3_ and 10%Ni-1%K/Al_2_O_3_. The 10%Ni-1%Ce/ZrO_2_ catalyst demonstrated an improved H_2_ yield and excellent catalytic performance. Further analysis revealed that the Ce promotor could store oxygen species and eliminate coke formation and sintering of the catalysts, resulting in its high performance. 

Su et al. [60] investigated the effects of La_2_O_3_ in promoting the Ni-La_2_O_3_/θ-Al_2_O_3_ catalyst in the SCWG of food waste. La enhanced the water–gas shift reaction, resulting in a high H_2_ yield. La also inhibited the methanation reaction, which is a major limitation of Ni-based catalysts. La also improved the metal dispersion, which increased the catalytic activity. Chowdhury et al. [61] also reported that Ni/Al_2_O_3_ with an La promoter can lead to excellent catalytic activity in the SCWG of food waste. Ni/9%La-Al_2_O_3_ showed high H_2_ and gas yields. La improved the mesoporous structure and increased the dispersion of Ni, which enhanced the water–gas shift reaction and increased the H_2_ yield. Ni/9%La-Al_2_O_3_ also demonstrated high stability, which could be attributed to its better anti-carbon deposition property.

Mastuli et al. [62] compared doped and supported Zn and Ni catalysts on MgO support in the SCWG of oil palm frond. The doped catalysts had high surface areas, high stability, and high-activity basic sites, resulting in high H_2_ yields compared to supported catalysts. Zn-based catalysts showed higher H_2_ yields than Ni-based catalysts for both supported and doped catalysts. Mastuli et al. [63] further investigated the structural and catalytic effects of Mg_1−x_Ni_x_O nanomaterial as a catalyst. They synthesized Mg_1−x_Ni_x_O nanomaterial via a self-propagating combustion method in the SCWG of oil palm frond. As the Ni content increased, the cell volume decreased linearly. This increased the specific surface area and improved the basic properties of the catalyst. The Mg_0.8_Ni_0.2_O catalyst with the highest Ni content demonstrated the highest gas and H_2_ yields. 

Li et al. [64] demonstrated that the formation of the char layer could be minimized using co-precipitated Ni-Mg-Al catalysts. They varied the Mg-Al molar ratio in the catalyst and investigated its effects in the SCWG of glucose. The catalysts favored H_2_ production, resulting in high H_2_ selectivity. Furthermore, Mg inhibited graphitic carbon formation because of its neutralizing action on alumina acidic sites, thus increasing the lifespan of the catalysts. However, the subsequent increase in Mg loading formed the MgNiO_2_ complex, which limited the activity of Ni metal.

Li et al. [65] also studied the stability and activities of various wet-impregnated Mg-promoted Ni catalysts on Al_2_O_3_ and CNT supports in the SCWG of glycerol. The stability studies showed the loss of Al, which resulted in deactivation of the Mg-promoted Ni-Al_2_O_3_ catalysts. Both the Ni/α-Al_2_O_3_ and Ni/γ-Al_2_O_3_ catalysts showed poorer stability and regenerability over repeated use than the Ni/CNT catalyst.

Li and Guo [66] compared the catalytic action of Mg-promoted Ni/Al_2_O_3_ catalysts synthesized via the co-precipitation and wet impregnation methods for a variety of feedstocks, such as glycerol, cellulose, glucose, poplar leaf, corncob, phenol, and sawdust. The results showed that the co-precipitated Ni-Mg-Al catalysts were more stable than the wet-impregnated Ni-Mg-Al catalysts. This was due to the growth of the crystal size of the wet-impregnated Ni-Mg-Al catalysts in SCW. Among different feedstocks, the co-precipitated Ni-Mg-Al catalysts were more active for the gasification of water-soluble organics as compared to real lignocellulosic biomasses.

**Figure 4:**
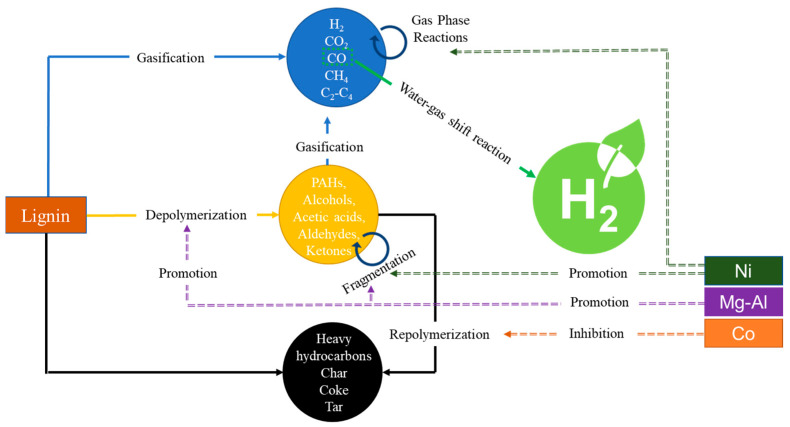
Catalytic mechanism of Ni-Co/Mg-Al in SCWG of lignin (adapted with permission from Kang et al. [67]).

Kang et al. [67] explored and proposed a detailed catalytic mechanism of Ni-Co supported on Mg-Al in the SCWG of lignin (Figure 4). The 2.6%Ni-5.2%Co/2.6%Mg-Al catalyst prepared via the co-precipitation method demonstrated high total gas and H_2_ yields due to significant improvement in its coke resistance ability. They also concluded that the co-precipitation method was more efficient than the wet-impregnated method. Norouzi et al. [68] showed that the addition of Ru on Fe-Ni/γ-Al_2_O_3_ could enhance gas yields while minimizing char formation. Another study by Lu et al. [50] showed that the addition of the Ce promoter on Ni/γ-Al_2_O_3_ was also capable of reducing coke and carbon deposition.

Catalysts synthesized in SCW have demonstrated high stability through their ability to reduce sintering. The supercritical water synthesis (SCWS) method for catalyst design provides better control over the size and shape of the nanoparticle without any requirement for organic solvents or precipitants. A few studies on SCWS synthesis of Ni-based catalysts on various supports (e.g., ZrO_2_, Ce-ZrO_2_, Al_2_O_3_, Mg-Al_2_O_3_, CNT and AC) have been reported for the SCWG of biomass [69,70]. SCWS-synthesized Ni/MgO-Al_2_O_3_ catalysts demonstrated the highest activities and stability. Despite their increased specific surface areas and pore volumes, SCWS-synthesized Ni/CeO_2_-ZrO_2_ catalysts showed no promotional effects when Ce was used. This was because of the low Ni particle dispersion in the Ni/CeO_2_-ZrO_2_ catalysts. However, as compared to sol-gel prepared catalysts, which have bigger bulk NiO particles, the SCWS-synthesized catalysts showed high dispersion and stable crystalline structures. After multiple use cycles, the SCWS-synthesized catalysts retained their high dispersion, whereas sol-gel-prepared catalysts experienced growth in size. This allowed the SCWS-prepared catalysts to maintain their high activities over repeated use, as opposed to catalysts prepared using conventional methods that may lose their activity over repeated use. Additionally, SCWS-synthesized catalysts are also synthesized in an environmentally friendly way as they do not require any organic solvents or robust chemical compounds. 

Li et al. [71] studied and proposed a catalytic mechanism in the SCWG of dewatered sewage sludge and various model compounds using AlCl_3_ combined with Ni, KOH, or K_2_CO_3_ catalysts and oxidants (e.g., H_2_O_2_, K_2_S_2_O_8_, and CaO_2_). AlCl_3_-H_2_O_2_ demonstrated the highest gas yields, followed by AlCl_3_-K_2_S_2_O_8_. AlCl_3_ combined with Ni, KOH, CaO, or K_2_CO_3_ catalysts resulted in low H_2_ yields as compared to AlCl_3_ alone. However, using K_2_S_2_O_8_ or H_2_O_2_ alone decreased the H_2_ yield. The H_2_ yield decreased, and gasification efficiency increased with a rise in the addition of oxidants. Interestingly, AlCl_3_-H_2_O_2_ (8:2) showed the highest gas yield, followed by AlCl_3_-K_2_S_2_O_8_ (8:2) and AlCl_3_. For the AlCl_3_-catalyzed SCWG of the model compound, glycerol resulted in the highest H_2_ yield, followed by guaiacol, glucose, alanine, and humic acid. Al_2_Cl_3_-H_2_O_2_ increased the H_2_ yield of humic acid by 17% but decreased the H_2_ yields of glucose and glycerol by 20% and 12%, respectively, compared to the AlCl_3_ catalyst. The authors also proposed a catalytic mechanism in the SCWG of dewatered sewage sludge with an AlCl_3_-H_2_O_2_ catalyst. They proposed that AlCl_3_ promoted the cleavage of the C–C bond with Al_3_^+^ ions. The Al_3_^+^ ions increased the acidity of SCW by reacting with water and forming Al(OH)_3_ and H^+^ ions. Al(OH)_3_ further underwent dehydration to form AlO(OH), which formed precipitates in water. The H^+^ and Cl^−^ ions enhanced the gasification of intermediate compounds to produce H_2_, thus increasing the H_2_ yield. H_2_O_2_ further enhanced the gasification of benzene-containing monomers by favoring the steam reforming reaction. In the case of sewage sludge, H^+^ generated via Al_3_^+^ deposition further enhanced the ring-opening activity of H_2_O_2_ to promote the decomposition of benzene-containing monomers into small molecules. These small organic molecules were further gasified by the combined catalytic effects of Cl^−^ and H^+^ ions to increase H_2_ yields.

Although Ni-based catalysts demonstrate improvement in gasification efficiency, they suffer from deactivation mainly because of tar formation and coke deposition [72]. Despite the high activity of Ni/γ-Al_2_O_3_-based catalysts, they still suffer from various issues, such as sintering, formation of Ni/Al_2_O_4_ complexes, and transformation of the γ-Al_2_O_3_ phase to the α-Al_2_O_3_ phase. These issues significantly hamper the catalysts’ stability. This is a severe issue for alumina-supported catalysts due to the ready conversion of intermediate products adsorbed on the acidic site into carbon, which deactivates Ni-based catalysts. The addition of alkali promoters can suppress cracking and polymerization reactions. Alkali promoters can also neutralize the acidic sites of alumina supports. Thus, alkali promotors can significantly reduce carbon formation.

#### 3.1.2. Ruthenium-Based Catalysts

Ru-based catalysts with promising metal dispersion are more reactive at low temperatures than Ni-based catalysts [73]. Ru-based catalysts have higher surface areas and distribution than Ni-based catalysts. Therefore, high surface area and more metal distribution can be achieved with relatively low Ru metal loading on the support material. Nguyen et al. [74] also confirmed that Ru-based catalysts show higher catalytic activities per metallic mass than Ni-based catalysts. Additionally, Ru-based catalysts are highly resistant to oxidation and hydrothermal conditions compared to Ni-based catalysts. Ru-based catalysts have higher activities toward hydrogenation and C–C bond cleavage [75]. When compared to other expensive transition metals, Ru-based catalysts exhibit the highest activity and H_2_ selectivity. 

As opposed to Ni-based catalysts, Ru-based catalysts are more susceptible to deactivation by sulfur poisoning [76]. To overcome sulfur sintering, a sacrificial agent with a relatively high affinity towards sulfur can be used to protect Ru from sulfur sintering. Peng et al. [77] used ZnO as a sacrificial agent with Ru/C catalysts to study the SCWG of microalgae (*Chlorella vulgaris*). ZnO showed high mechanical stability and sulfur adoption performance, which minimized Ru metal sintering. Despite Ru-based catalysts having high surface areas, high dispersion, and high catalytic performance, the relatively low cost of Ni-based catalysts makes them preferable for large-scale industrial applications over Ru-based catalysts.

Kang et al. [29] also observed that Ru/Al_2_O_3_ showed the highest metal dispersion compared to Ni-based catalysts. They concluded that 5%Ru/Al_2_O_3_ demonstrated a higher H_2_ yield than the 5%Ni/Al_2_O_3_ catalyst in the SCWG of cellulose and lignin. Therefore, for the same metal loading, Ru-based catalysts had higher H_2_ yields than Ni-based catalysts. Nanda et al. [55] compared Ru/Al_2_O_3_ with Ni/Si-Al_2_O_3_, K_2_CO_3_, and Na_2_CO_3_ catalysts in the SCWG of waste cooking oil. The order of H_2_ yield was Ru/Al_2_O_3_ > Ni/Si-Al_2_O_3_ > K_2_CO_3_ > Na_2_CO_3_. The effects of metal loading showed that 5 wt% Ru/Al_2_O_3_ resulted in the maximum H_2_ yield.

The superior catalytic performance of Ru/Al_2_O_3_ catalysts has also been reported in the SCWG of glucose and guaiacol [75,78]. In the SCWG of glucose, the Ru/Al_2_O_3_ catalyst inhibited the production of furfural and 5-hydroxymethylfurfural while favoring the degradation of intermediates such as phenols, ketones, acids, and arenes [75]. Enhanced gasification of intermediates improved process efficiency and increased total gas and H_2_ yields while preventing the formation of char. During the SCWG of guaiacol, Ru/Al_2_O_3_ catalysts enhanced the conversion of phenol to cyclohexanol by favoring the hydrogenation reaction and the conversion of cyclohexanol to hexanone or hexenol by favoring ring-opening reactions [78]. Hexanone and hexenol can further decompose into small gaseous molecules, including H_2_. Thus, Ru/Al_2_O_3_ improved H_2_ and total gas yields while minimizing char and tar formation. 

Zhang et al. [58] observed the effects of Ni and Ru bimetallic catalysts supported on γ-Al_2_O_3_. They recommended the use of Ni and Ru bimetallic catalysts supported on γ-Al_2_O_3_ in the SCWG of glucose to achieve high activity and H_2_ selectivity. Hossain et al. [52] further investigated various bimetallic Ni-Ru/Al_2_O_3_-supported aerogel catalysts. Ni-Ru/Al_2_O_3_ aerogel catalysts demonstrated 1.3- and 1.6-times higher H_2_ yields than mesoporous and wet-impregnated synthesized Ni-Ru/Al_2_O_3_ catalysts for the same amount of metal loading. The aerogel catalysts showed high and uniform metal particle dispersion with strong interaction between the support and active metal. The high catalytic performance of the aerogel catalysts was due to the supercritical CO_2_ drying step during aerogel catalyst synthesis, which improved the surface area and reactant diffusivity. A significant decrease in coke formation was also observed with the aerogel catalysts due to their low acidity. This resulted in high stability and activities of the aerogel catalysts. 

Tushar et al. [79] confirmed the catalytic effects of Ni and Ru catalysts. They investigated ten different combinations of Ni and Ru catalysts on various supports, such as γ-Al_2_O_3_ and ZrO_2_. Overall, Ni-Ru/γ-Al_2_O_3_-ZrO_2_ demonstrated the maximum H_2_ yields and high carbon gasification efficiency. Ni-Ru/γ-Al_2_O_3_-ZrO_2_ also demonstrated high stability and activities over repeated use. In another study, dual-component catalysts having equal amounts of Ru/C-Ru/C demonstrated better catalytic activities than single-component catalysts [80]. 

Yang et al. [81] investigated the kinetics and intermediate products of Ni-Ru/Al_2_O_3_ bimetallic catalysts for the SCWG of phenol. They proposed that phenol converted into an enol intermediate via a partial hydrogenation reaction. Furthermore, enol rapidly formed cyclohexanone. This observation was different from the mechanism proposed by Zhu et al. [78] where cyclohexanone was considered as an intermediate product for the formation of cyclohexanol. The kinetic study revealed that phenol was more difficult to gasify than the intermediate compounds. Interestingly, steam reforming of cyclohexanone was not the main contributor to H_2_ production due to its lower concentration than phenol.

#### 3.1.3. Other Heterogeneous Catalysts

Apart from Ni and Ru, other transition metals such as Pt, Co, and Rh (supported or unsupported) are also used as heterogeneous catalysts in the SCWG process. Karakuş et al. [49] investigated Pt/Al_2_O_3_ and Ru/Al_2_O_3_ catalysts in the SCWG of 2-propanol. Their results showed that the H_2_ selectivity of Pt/Al_2_O_3_ was relatively higher than that of Ru/Al_2_O_3_ due to enhancement of the methanation reaction, which produced CH_4_ at the expense of H_2_. Pairojpiriyakul et al. [82] used Co-based catalysts on a variety of supports, such as α-Al_2_O_3_, ZrO_2_, γ-Al_2_O_3_, La_2_O_3_, and yttria-stabilized zirconia (YSZ), in the SCWG of glycerol. The highest H_2_ yield was obtained with Co/YSZ. In addition, increasing the Co loading up to 10% improved the gasification efficiency of glycerol and H_2_ production. However, a further increase in the Co loading decreased both H_2_ yield and glycerol conversion. 

Deactivation, sintering, and poisoning of heterogeneous catalysts by sulfur or coke is still a major challenge. Additionally, heterogeneous catalysts oxidize the elemental sulfur and chlorine in biomass to acids. Retention of these acids in the liquid products of SCWG poses a serious challenge for its disposal and/or recycling. The non-polar nature of SCW dissolves the organic compounds during hydrothermal gasification but the inorganic components, including the active metal (catalyst) and mineral matter (catalyst support), can precipitate and form agglomerates in the reactor if not removed properly. The gradual deposition of these precipitates and agglomerates can corrode the reactor during high-temperature and high-pressure operations [83]. Nevertheless, more advancements are needed to address these challenges to synthesize suitable heterogeneous catalysts with high activity, regenerability, and stability, with resistance to sintering and deactivation. 

### 3.2. Metal Oxide Catalysts

Metal oxide catalysts are rarely used in the SCWG process and very little literature is available on their catalytic performance in SCWG processes. They are generally used as supports to improve the stability and activities of metal-supported catalysts. The most common metal oxides used in SCWG processes are RuO_2_, ZrO_2_, and CeO_2_. Cao et al. [84] compared different metal oxides catalysts such as V_2_O_5_, MnO_2_, Cr_2_O_3_, Fe_2_O_3_, CuO, Co_2_O_3_, ZnO, MoO_3_, ZrO_2_, SnO_2_, CeO_2_, and WO_3_ in SCWG of glucose. Among all metal oxide catalysts, Cr_2_O_3_, CuO, and WO_3_ showed high gasification efficiencies compared to Fe_2_O_3_, ZnO, and ZrO_2_. The H_2_ yields decreased with almost all metal oxide catalysts, except Cr_2_O_3_, which improved the H_2_ yield. 

**Figure 5:**
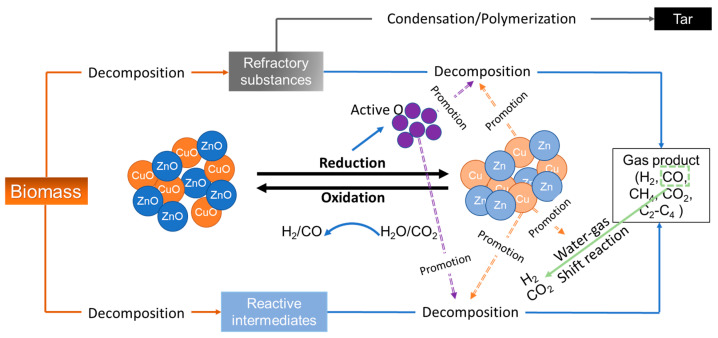
Catalytic mechanism of CuO-ZnO in SCWG of biomass (adapted with permission from Cao et al. [85]).

Various co-precipitated binary metal oxide catalysts, such as CeO_2_-ZrO_2_, CuO-ZnO, and Fe_2_O_3_-Cr_2_O_3_, have demonstrated high catalytic performance in SCWG [85,86]. Cao et al. [85] showed that in the SCWG of lignin, the CuO-ZnO catalyst demonstrated high catalytic performance with a high H_2_ yield and better gasification efficiency, followed by Fe_2_O_3_-Cr_2_O_3_ and CeO_2_-ZrO_2_. However, in the SCWG of cellulose, Fe_2_O_3_-Cr_2_O_3_ showed a greater H_2_ yield and high carbon gasification efficiency, followed by CuO-ZnO and CeO_2_-ZrO_2_. This was due to the higher oxygen content of cellulose compared to lignin. Thus, oxygen released by metal oxide catalysts had less pronounced effects in the SCWG of cellulose. Additionally, the H_2_ yield from cellulose was less than that from lignin, which also decreased the reducibility of the reaction medium. The catalytic mechanism of binary metal oxide catalysts showed that CeO_2_ was the main active component in the CeO_2_-ZrO_2_ catalyst [86]. CeO_2_ distributed on ZrO_2_ released active oxygen via redox reactions to enhance the SCWG process. ZrO_2_ also absorbed active H_2_ and small intermediates to increase contact between the intermediates and CeO_2_ for improved catalytic performance. In CuO-ZnO, Cu was the main active component, which released oxygen species (Figure 5). ZnO acted as a structural stabilizer, promotor and absorbent for sulfur in the CuO-ZnO supported catalyst. 

Onwudili [87] studied the detailed catalytic mechanism of RuO_2_/γ-Al_2_O_3_ in the SCWG of municipal solid waste. RuO_2_/γ-Al_2_O_3_ drastically increased H_2_, CH_4_, and CO_2_ yields while significantly improving gasification efficiency. The high yield of H_2_ was due to enhancement of the water–gas shift reaction by the catalytic action of RuO_2_/γ-Al_2_O_3_. In addition, the enhancement of methanation of CO or CO_2_ and hydrogenolysis of C–C hydrocarbons resulted in a high CH_4_ yield. Improvement in the yields of the reduction product (CH_4_) and oxidation product (CO_2_) indicated the involvement of the RuO_2_/γ-Al_2_O_3_ catalyst in Ru(IV) and Ru(0) cyclic redox reactions. Reduction of Ru(IV) into Ru(0) was essential for the SCWG process, whereas oxidation of Ru(0) into Ru(IV) was necessary for the catalytic process. The primary synergetic effects were due to the improvement of the dispersion of RuO_2_ on γ-Al_2_O_3_, which resulted in enhanced carbon gasification efficiency. 

Samiee-Zafarghandi et al. [88] compared MnO_2_/SiO_2_ and NiO/SiO_2_ catalysts in the SCWG of microalgae *Chlorella*. MnO_2_/SiO_2_ demonstrated the highest H_2_ yield (1.1 mmol/g) compared to NiO/SiO_2_ (0.6 mmol/g) and non-catalytic SCWG (0.2 mmol/g). Therefore, NiO/SiO_2_ was less active than the supported MnO_2_/SiO_2_. Borges et al. [89] investigated the Ni/Fe_2_O_4_ catalyst in the SCWG of *Eucalyptus* wood chips. Ni/Fe_2_O_4_ enhanced the H_2_ yield and decreased the char yield. Further investigation showed that Ni/Fe_2_O_4_ favored the water–gas shift and steam reforming reactions, thus increasing H_2_ yield and decreasing CH_4_ yield. It also demonstrated good stability and recyclability despite the coke deposit [90].

## 4. Novel Carbon-Based Catalysts Used in Hydrothermal Gasification

Carbon-based supports can also be used with transition metals in the SCWG of biomass. Their high surface areas along with the renewable and biodegradable nature of activated carbon and other carbon-based supports make them sustainable catalytic materials. Table 3 summarizes some notable studies on the use of carbon-based catalysts for SCWG processes [65,71,91,92,93,94]. Taylor et al. [95] compared Ni/AC and Ru/AC with other catalysts such as KOH, Trona, dolomite, and Borax in the SCWG of wood chips. Both Ni/AC and Ru/AC demonstrated higher H_2_ yields because of improved water–gas shift compared to other non-carbonaceous catalysts.

Yamaguchi et al. [96] investigated various metals (e.g., Ru, Ni, Pt, Rh and Pd) supported on activated carbon in the SCWG of woody biomass. The Ru/AC catalysts demonstrated the highest gas yields, followed by Rh/AC, Pt/AC, Pd/AC, and Ni/AC. Ru/AC showed the highest activity for lignin gasification. However, it showed an inferior H_2_ yield, which was due to enhancement of the methanation reaction, which consumed H_2_. Interestingly, the Pd/AC catalyst demonstrated the highest H_2_ yield, followed by Ru/AC, Pt/AC, Rh/AC, and Ni/AC. Thus, Pd/AC showed the best H_2_ yield but poor gas yields, whereas Ni/AC showed the lowest gas and H_2_ yields. Activated carbon also improved the H_2_ yield over a wide range of reaction temperatures. 

Osada et al. [94] investigated TiO_2_ and activated carbon as supports for Ru catalysts in the SCWG of lignin, cellulose, and sugarcane bagasse. Ru/AC demonstrated the highest gasification efficiency with near-complete gasification of sugarcane bagasse in 15 min. For the same amount of Ru metal, Ru/AC showed slightly higher activity as compared to Ru/TiO_2_ catalysts. This was due to the high Ru metal dispersion of 51% in the Ru/AC catalyst as compared to 27% metal dispersion in Ru/TiO_2_. However, the gas yield and composition of both catalysts were the same when 100% carbon conversion was achieved. This indicated that the equilibrium gas yield and composition did not have any correlation with metal dispersion. For the Ru/AC catalysts, repetitive use increased H_2_ selectivity but decreased CH_4_ selectivity due to disintegration of the active sites for the methanation reaction. However, Ru/AC suffered from deactivation since its activity decreased significantly after repetitive use. Therefore, more active and durable AC-based catalysts need to be developed to overcome these challenges. Yamaguchi et al. [96] reported that Ru/γ-Al_2_O_3_ demonstrated high gasification activity but low stability as the crystallographic phase of γ-Al_2_O_3_ transformed into α-Al_2_O_3_. 

CNT is another carbon-based support that has a large surface area, high heat conductivity, excellent chemical and physical stability, and a tunable porous structure. Among SCWS-prepared metal-impregnated carbon catalysts, CNT-based catalysts showed higher activities and stability than active carbon and Al_2_O_3_ supported catalysts [97]. At reaction conditions of 480 °C, 25 MPa, and 10–50 h, Ni/CNT resulted in the highest H_2_, CO, CH_4_, and total gas yields, followed by Ni/AC, Ni/Al_2_O_3_, and Ni catalysts. Ni/CNT maintained its high activity even at a longer reaction time of 50 h, whereas Ni/AC and Ni/Al_2_O_3_ significantly dropped their activities after 30 h of use. This was primarily due to the leaching of active Ni metal in the Ni/AC and Ni/Al_2_O_3_ catalysts. 

Rashidi and Tavasoli [98] evaluated the effects of a copper promoter on Ni/CNT catalysts in the SCWG of sugarcane bagasse. Cu-promoted Ni/CNT was found to increase the H_2_ and total gas yields but decreased the CH_4_ yield. Thus, Cu-promoted Ni/CNT catalysts overcome the methanation tendency of Ni, which is a major limiting factor of Ni-based catalysts. Azadi et al. [28] reported that Ni-Cu/CNT showed a nearly ten-fold increase in H_2_ yield and 40 times less CH_4_ yield with a significant reduction in CO_2_ yield. Thus, Cu-promoted Ni/CNT catalysts have high H_2_ selectivity and low CH_4_ and CO_2_ selectivities. Li et al. [65] confirmed the high catalytic stability over repeated use of Ni/CNT catalysts in the SCWG of glycerol. de Vlieger et al. [99] also showed the high stability of Pd/CNT catalysts in the SCWG of ethylene glycerol. Pt/CNT exhibited no mass loss with no change in the size and distribution of Pt particles on CNT during SCWG. 

Carbonaceous materials such as hydrochar and biochar are also potential materials for the development of catalysts. Safari et al. [91] investigated the performance of catalysts developed from the hydochars of green algae (*Cladophora glomerata*) and wheat straw in the SCWG of almond shells. The high amounts of alkali and alkaline earth metals in the hydrochar samples enhanced the cracking of biopolymers and favored the water–gas shift reaction, thus increasing the H_2_ yield. The total gas yield and H_2_ fraction were selectively improved from 26.7 mmol/g and 41% in the non-catalytic run to 29.2 mmol/g and 58%, respectively, when wheat straw hydrochar was used as the catalyst. The total gas yield and H_2_ concentration also increased to 31.1 mmol/g and 60%, respectively, when green algae hydrochar was used as the catalyst in the SCWG of almond shells. 

Another novel method for catalytic SCWG is the in-situ impregnation of metal nanoparticles in biomass feedstock. This approach can overcome the issue of deactivation encountered by conventional catalysts and help to reduce the cost of catalyst preparation. Nanda et al. [93] carried out the SCWG of pinewood and wheat straw impregnated with Ni-nanoparticles. Ni-impregnated biomasses demonstrated high H_2_, CO_2_, and CH_4_ yields compared to the raw feedstocks. Huang et al. [100] also used in-situ-generated Ni particles using nickel acetate as a precursor for the gasification of glucose in SCW. In-situ-generated Ni catalysts demonstrated superior catalytic performance compared to nickel wire catalysts. They also proved the role of in-situ generated Ni particles from nickel acetate in enabling the catalytic production of H_2_ during SCWG of glucose. 

Kumar and Reddy [101] investigated the SCWG of in-situ Ni-impregnated sugarcane bagasse and lemon peels and compared the results with the Raney nickel catalyst. They used nickel nitrate hexahydrate salt as a precursor for the in-situ generation of nickel nanoparticles. Both Ni-impregnated biomasses demonstrated significantly higher gas yields, H_2_ yields, and carbon gasification efficiencies than Raney nickel. Ni-impregnated sugarcane bagasse achieved higher carbon gasification efficiency, gas yield, and H_2_ yield than Ni-lemon peel. Kumar and Reddy [92] also performed the SCWG of banana pseudo-stem using impregnation of Ni, Ru, and Fe metals onto the biomass as the support material. The H_2_ yields and gasification efficiencies of the metals were in order of Ni > Ru > Fe. The superior performance of Ni to act as an in-situ nanocatalyst is due to its ability to effectively cleave C–H and C–C bonds for improved reforming reactions [102]. However, very little literature is available on the development of in-situ nanocatalysts impregnated onto biomass for proper assessment of their robustness, stability, regeneration, and post-gasification compared to commercially available homogeneous and heterogeneous catalysts. One of the limitations in the design of such novel catalysts can be the presence of lignin and other mineral matter in the biomass [103,104], which can hinder the penetration of catalytic nanoparticles within the cell wall. Therefore, more research is needed for a better understanding of such catalysts and to address these limitations.

## 5. Conclusions and Perspectives

SCWG is a promising technology for the sustainable production of H_2_ due to its various advantages over other thermochemical processes. SCWG has shown its potential for converting a wide variety of low-value biomasses into high-value H_2_-rich gas products. This can serve as a green alternative to the steam methane reforming process due to the renewable and clean nature of biomass sources compared to fossil fuels. However, SCWG requires high energy input to achieve supercritical conditions. Nonetheless, catalysts are used to achieve high gas yields and process efficiencies even at near-critical conditions. 

Various homogeneous and heterogeneous catalysts have been studied to achieve high H_2_ yields at low temperatures in SCWG processes. Although homogeneous catalysts are suitable compared to heterogeneous catalysts, they suffer from recovery issues. This also increases the cost of the process and hinders its use in large-scale industrial applications. On the other hand, heterogeneous catalysts are relatively easier to recover, but they can suffer from deactivation. Deactivation of heterogeneous catalysts can occur for various reasons, such as fouling, poising, sintering, and char formation. Transition metal catalysts (e.g., Ni, Cu, Co, and Ru) have demonstrated excellent performance in enhancing SCWG reactions. Ru- and Ni-based catalysts are the most widely used catalysts owing to their superior performance in SCWG processes, especially in water–gas shift, hydrogenation, and methanation reactions. Novel catalysts such as activated carbon, char, CNT, and lignocellulosic biomass impregnated with catalytic nanoparticles have demonstrated promising potential to achieve comparable catalytic performance and renewability in SCWG reactions. 

It cannot be denied that SCWG is an innovative and viable technology for producing combustible gases with higher selectivity to individual gas components using catalysts. However, a detailed study of the economic viability and technical feasibility of these catalysts is needed. New developments in the field of catalysts can facilitate the commercialization of SCWG technology. Extensive research strategies are required to tackle the unique challenges faced by SCWG technology that prevents its scalability and commercialization. Some of the common challenges are associated with reactor corrosion, plugging due to salt and mineral precipitation, the requirement of special reactor set-up resistant to high temperatures, high pressures, and corrosion, coking of catalyst supports, as well as catalyst poisoning, sintering, and deactivation. The techno-economic, environmental, and lifecycle viability of SCWG technology on a commercial scale is also contingent on the efficient conversion of feedstocks, catalyst recovery, regeneration and reuse, effective separation of gas, liquid, and solid products, as well as upgrading and applications of main products and co-products. Nonetheless, SCWG remains an appealing technology with many benefits in the use of water as a source of aqueous reaction media to valorize complex feedstocks and pollutants under environmentally benign conditions while addressing the issues of waste management and clean energy recovery.

## Figures and Tables

**Table 1:** Notable studies on SCWG of waste biomass assisted by homogeneous catalysts.

Feedstock	Catalyst	Operating Conditions	Main Findings	Reference
Cellulose and lignin	K_2_CO_3_	Temperature: 300–600 °C Reaction time: 1 h Feed concentration: 7.4 wt% (0.45 M) Pressure: 9–41 MPa Reactor: Batch	K_2_CO_3_ enhanced gasification efficiency and limited char formation. The highest H_2_ and CH_4_ yields were obtained with K_2_CO_3_ at 600 °C.	Kang et al. [29]
Cellulose and lignin	K_2_CO_3_	Temperature: 300–600 °C Reaction time: 1 h Pressure: 9–41 MPa Reactor: Batch	K_2_CO_3_ enhanced the water–gas shift reaction, leading to a high H_2_ yield. The highest H_2_ yield of 28 mmol/g was achieved with K_2_CO_3_ at 600 °C from SCWG of glucose. Total organic carbon levels decreased with catalyst loading, indicating efficient gasification of the feedstock.	Madenoğlu et al. [30]
Glucose	KOH	Temperature: 450–560 °C Reaction time: 6–10 s Feed concentration: 0.2–2 wt% Pressure: 25 MPa Reactor: Continuous	The highest heating value of 113% was achieved with KOH catalysts at optimized gasification conditions.	Garcia-Jarana et al. [31]
Glucose	Raney nickel and K_2_CO_3_	Temperature: 500 °C Reaction time: 1 h Feed concentration: 5 wt% Pressure: 30 MPa Reactor: Batch	0.5 wt% K_2_CO_3_ demonstrated better catalytic activity than 1 wt% Raney nickel.	Sınaǧ et al. [32]
Paper sludge and black liquor	KOH, K_2_CO_3_, and NaOH	Temperature: 500–650 °C Reaction time: 2 min Feed concentration: 2–3 wt% Pressure: 25 MPa Reactor: Semi-continuous	The highest H_2_ yield of 25 mmol/g was obtained with K_2_CO_3_ at 600 °C from SCWG of paper sludge.	Rönnlund et al. [33]
Sewage sludge	KOH, K_2_CO_3_, NaOH, Na_2_CO_3_, and AC	Temperature: 450 °C Reaction time: 1 h Pressure: 23–26 MPa Reactor: Batch	KOH increased gas yield to 12.2 mmol/g from 11.3 mmol/g in the non-catalytic run. K_2_CO_3_ demonstrated the highest desulfurization effect followed by Na_2_CO_3_, NaOH, KOH, and AC.	Feng et al. [34]
Timothy grass	KOH, K_2_CO_3_, NaOH, and Na_2_CO_3_	Temperature: 650 °C Reaction time: 45 min Biomass-to-water ratio: 1:8 Pressure: 23–25 MPa Reactor: Batch	KOH demonstrated the highest H_2_ yield (8.9 mmol/g) followed by K_2_CO_3_ (7.8 mmol/g), NaOH (6.7 mmol/g), and Na_2_CO_3_ (6.3 mmol/g). KOH enhanced the water–gas shift reaction to maximize the H_2_ yield. NaOH enhanced the methanation reaction favoring CH_4_ formation at the expense of H_2_.	Nanda et al. [35]

**Table 2:** Notable studies on SCWG of waste biomass assisted by heterogeneous catalysts.

Feedstock	Catalyst	Operating Conditions	Main Findings	Reference
2-Propanol	Pt/Al_2_O_3_ and Ru/Al_2_O_3_	Temperature: 400–550 °C Reaction time: 10–30 s Feed concentration: 0.5 M Pressure: 25 MPa Reactor: Continuous	Pt/Al_2_O_3_ showed high H_2_ selectivity at lower temperatures than Ru/Al_2_O_3_. Ru/Al_2_O_3_ showed 10 mol% H_2_ compared to 96 mol% H_2_ in the case of Pt/Al_2_O_3_. The low H_2_ selectivity of Ru/Al_2_O_3_ was due to enhancement of the methanation reaction, which led to CH_4_ yields.	Karakuş et al. [49]
Glucose	Ni/Al_2_O_3_ and Ni/CeO_2_-Al_2_O_3_	Temperature: 400 °C Feed concentration: 9.1 wt% Pressure: 24.5 MPa Reactor: Batch	Both catalysts significantly improved the H_2_ yield and selectivity. Ni/CeO_2_-Al_2_O_3_ showed superior catalytic activity than Ni/Al_2_O_3_ with higher yields of total gases and H_2_. The high activity of Ni/CeO_2_-Al_2_O_3_ was attributed to the inhibition of coke formation and sintering by Ce metal in catalysts. Further addition of Ce improved the H_2_ yield and selectivity, attaining maxima at 8.5 wt% loading.	Lu et al. [50]; Lu et al. [51]
Glucose	Ni/Al_2_O_3_ and Ru-Ni/Al_2_O_3_	Temperature: 400–500 °C Feed concentration: 45 kg/m^3^ Pressure: 25–35 MPa Reactor: Batch	Aerogel-synthesized catalysts showed high H_2_ yields compared to mesoporous and wet-impregnated catalysts. The supercritical CO_2_ drying step in the aerogel synthesis method enhanced the surface area and reactant diffusivity to improve catalytic performance. Ru-Ni/Al_2_O_3_ demonstrated the highest H_2_ yield of 4.9 mmol/g. The high H_2_ yield and stability of Ru-Ni/Al_2_O_3_ were due to the inhibition of graphite coke formation by Ru metal.	Hossain et al. [52]
Glucose, cellulose, fructose, xylan, pulp, lignin, and bark	Ni/Al_2_O_3_, Ni/hydrotalcite, Raney nickel, Ru/C, and Ru/Al_2_O_3_	Temperature: 380 °C Reaction time: 15 min Feed concentration: 2 wt% Pressure: 25 MPa Reactor: Batch	Ni/Al_2_O_3_ demonstrated the highest H_2_ selectivity. Ni/hydrotalcite showed the highest H_2_ yield for all the feedstocks followed by Ni/Al_2_O_3_. The high H_2_ yield of Ni/hydrotalcite and Ni/Al_2_O_3_ was attributed to the poor dispersion of Ni metal.	Azadi et al. [27]
Plastic wastes	NiO/γ-Al_2_O_3_, RuO_2_/γ-Al_2_O_3_, and bimetallic catalysts	Temperature: 450 °C Reaction time: 1 h Feed concentration: 20 wt% Pressure: 25 MPa Reactor: Batch	The highest carbon gasification efficiency of 99% was achieved with polypropylene followed by high-density polyethylene, low-density polyethylene, and polystyrene. The highest H_2_ yield in the non-catalytic run was achieved with low-density polyethylene followed by polystyrene, polypropylene, and high-density polyethylene. Compared to only using NiO, the bimetallic catalyst with RuO_2_ increased the H_2_ yield and reduced C_2_–C_4_ gas yields.	Onwudili and Williams [53]
Soyabean straw	Ni supported on carbon nanotubes (CNT), ZrO_2_, Al_2_O_3_, SiO_2_, and Al_2_O_3_-SiO_2_, and promoted by K, Ce, and Na.	Temperature: 500 °C Reaction time: 45 min Biomass-to-water ratio: 1:10 Pressure: 23–25 MPa Reactor: Batch	Ni supported on ZrO_2_ and Al_2_O_3_ demonstrated superior performance compared to other supports. 10%Ni-1%Ce/ZrO_2_ showed the highest H_2_ yield of 10.9 mmol/g and excellent catalytic performance. This was attributed to the high oxygen storage and mobility capabilities of Ce promotors for high reduction and oxidation performance.	Okolie et al. [54]
Waste cooking oil	Ru/Al_2_O_3_, Ni/Si-Al_2_O_3_, K_2_CO_3_, and Na_2_CO_3_	Temperature: 375–675 °C Reaction time: 15–60 min Feed concentration: 25–40 wt% Pressure: 23–25 MPa Reactor: Batch	The order of catalytic performance in enhancing the H_2_ yield was Ru/Al_2_O_3_ (10.2 mmol/g) > Ni/Si-Al_2_O_3_ (9.3 mmol/g) > K_2_CO_3_ (8.1 mmol/g) > Na_2_CO_3_ (7.5 mmol/g). Ru enhanced the water–gas shift reaction to improve H_2_ yields.	Nanda et al. [55]
Wheat straw	Ni/MgO, Fe/MgO, Cu/MgO, Ni/ZnO, Ni/Al_2_O_3_, and Ni/ZrO_2_	Temperature: 450 °C Reaction time: 20 min Feed concentration: 7.4 wt% Pressure: 23–28 MPa Reactor: Batch	The order of H_2_ yield was Ni/MgO (11.6 mmol/g) > Fe/MgO (9.2 mmol/g) > Cu/MgO (8.1 mmol/g). Among Ni-based supported catalysts, Ni/MgO demonstrated the highest H_2_ yields. Basic supports favored water–gas shift reactions, leading to high H_2_ yields.	Lu et al. [51]

**Table 3:** Notable studies on SCWG of waste biomass assisted by novel carbonaceous catalysts.

Feedstock	Catalyst	Operating Conditions	Main Findings	Reference
Almond shell	Hydrochar generated from SCWG of wheat straw and algae (*Cladophora glomerata*)	Temperature: 460 °C Reaction time: 10 min Feed-to-water ratio: 0.01 Pressure: 25 MPa Reactor: Batch	Hydrochar from algal and wheat straw demonstrated H_2_ yields of 11.6 and 10.8 mmol/g, respectively. Algal hydrochar showed higher H_2_ yield, H_2_ selectivity, and total gas yield due to the presence of alkali and alkaline earth metals, which enhanced the water–gas shift reaction.	Safari et al. [91]
Banana pseudo-stem	In-situ impregnated biomass with Fe, Ru, and Ni	Temperature: 600 °C Reaction time: 1 h Pressure: 22–25 MPa Reactor: Batch	Impregnated metals dramatically improved gasification efficiency and H_2_ yields. Ni-impregnated biomass showed the highest H_2_ yield (11.1 mmol/g) followed by Ru (8.8 mmol/g) and Fe (4.2 mmol/g). Ni nanoparticles enhanced cracking and reforming reactions. Ru nanoparticles favored the methanation reaction. Fe nanoparticles formed an oxide layer and promoted H_2_ yields.	Kumar et al. [92]
Dewater sewage sludge and model compounds	AlCl_3_ with Ni, KOH, and K_2_CO_3_ catalysts with H_2_O_2_, K_2_S_2_O_8_, and CaO_2_ oxidants	Temperature: 400 °C Reaction time: 30 min Feed concentration: 5 wt% Pressure: 24 MPa Reactor: Batch	AlCl_3_-K_2_CO_3_ led to the highest H_2_ yield (0.85 mmol/g) followed by Ni (0.8 mmol/g) and KOH (0.7 mmol/g), compared to using AlCl_3_ alone (7.8 mmol/g). Oxidants performed better than catalysts. AlCl_3_ combined with H_2_O_2_ showed the highest H_2_ yield of 8.9 mmol/g. However, oxidants alone decreased the H_2_ yield. Glycerol demonstrated the highest H_2_ yield followed by guaiacol, glucose, alanine, and humic acid with AlCl_3_.	Li et al. [71]
Glycerol	Ni/MgAl_2_O_4_-Al_2_O_3_, Ni/Al_2_O_3_, and Ni/CNT	Temperature: 425 °C Reaction time: 54 s Feed concentration: 5 wt% Pressure: 25.2 MPa Reactor: Continuous	Ni/CNT demonstrated high stability with superior carbon gasification efficiency. Ni/CNT improved the H_2_ yield by 2.7 times compared to the non-catalytic SCWG. Ni/MgAl_2_O_4_ showed the highest H_2_ yield (1.5 mol/mol) followed by Ni/Al_2_O_3_ (1.0 mol/mol) and Ni/CNT (0.8 mol/mol).	Li et al. [65]
Pinewood and wheat straw	In-situ Ni impregnation	Temperature: 500 °C Reaction time: 45 min Pressure: 23–25 MPa Reactor: Batch	In-situ Ni-impregnated pinewood showed improvements of 59%, 40%, and 34% in H_2_ yield, total gas yield, and carbon gasification efficiency, respectively, compared to non-catalytic run. Ni impregnation resulted in an H_2_ yield of 5.8 mmol/g from wheat straw and 2.8 mmol/g with pinewood. High H_2_ yield with wheat straw was attributed to a better distribution of Ni nanoparticles in wheat straw.	Nanda et al. [93]
Sugarcane bagasse	Ru/AC and Ru/TiO_2_	Temperature: 400 °C Reaction time: 15 min Pressure: 22–25 MPa Reactor: Batch	Ru/AC demonstrated superior activity compared to Ru/TiO_2_ with near-complete gasification of sugarcane bagasse in 15 min compared to 30 min using Ru/TiO_2_. H_2_ yield increased with repeated use of Ru/AC catalyst but it suffered from deactivation leading to a significant reduction in its activity.	Osada et al. [94]

## Data Availability

Not applicable.
